# Transition from paediatric to adult congenital heart disease care in Central and South-Eastern Europe: current practices and opportunities for improvement

**DOI:** 10.1093/ehjopen/oeag144

**Published:** 2026-09-26

**Authors:** Alexandra Arvanitaki, Katja Prokšelj, Iveta Šimkova, Irena Ivanac Vranešić, Agnese Strenge, Monika Komar, Magdalena Lipczynska, Anna Kaneva, Lina Gumbiene, Petra Antonova, Hajnalka Olga Balint, Ioan Mircea Coman, Umit Yasar Sinan, Maryanne Caruana, Aleksandra Nikolic, Maarja Maser, Elena Panfile, Margarita Bartsota, Elizabeta Srbinovska Kostovska, George Giannakoulas, Tamara Kovačević-Preradović, Gani Bajraktari, Pencho Kratunkov, Lidia Tomkiewicz-Pajak, Yuliia Klymyshyn, Ihor Lebid, Michael A Gatzoulis, Margarita Brida

**Affiliations:** 1st Cardiology Department, School of Medicine, Faculty of Health Sciences, Aristotle University of Thessaloniki, St. Kyriakidi 1, Thessaloniki 54124, Greece; 2nd Cardiology Department, School of Medicine, Faculty of Health Sciences, Aristotle University of Thessaloniki, Konstantinoupoleos 52, Thessaloniki 54641, Greece; Adult Congenital Heart Centre and National Centre for Pulmonary Hypertension, Royal Brompton & Harefield Hospitals, Guys & St Thomas’s NHS Trust, Sydney Street, London SW3 6N5, UK; Department of Cardiology, University Medical Centre Ljubljana and Faculty of Medicine, University of Ljubljana, Zaloska St. 2, Ljubljana 1000, Slovenia; Department of Cardiology and Angiology, Adult Congenital Heart Disease Division, Slovak Medical University, National Institute of Cardiovascular Diseases, Pod Krasnou Horkou 1/7185, Bratislava 83348, Slovakia; Department of Cardiovascular Diseases, University Hospital Center Zagreb, Kispaticeva 12, Zagreb 10000, Croatia; Latvian Center of Cardiology, Pauls Stradins Clinical University Hospital, 13 Pilsonu Street, Riga LV-1002, Latvia; Department of Cardiac and Vascular Diseases, Institute of Cardiology, Jagiellonian University Medical College, John Paul II Hospital, Pradnicka 80, Kraków 31-202, Poland; Department of Congenital Heart Diseases, National Institute of Cardiology, Alpejska 42, Warsaw 04-628, Poland; Department of Heart, Chest and Transplant Surgery, University Clinical Center, Medical University of Warsaw, Stefana Banacka 1A, Warsaw 02-097, Poland; National Heart Hospital, Konyovitsa Street 65, Sofia 1309, Bulgaria; Clinic for Cardiac and Vascular Diseases, Faculty of Medicine, Vilnius University Hospital, Santariskiu 2, Vilnius 08661, Lithuania; Cardiovascular Centre, University Hospital Motol, Uvalu 84, Prague 15006, Czech Republic; Gottsegen National Cardiovascular Center, Haller Street 29, Budapest 1096, Hungary; Institutul de Urgență Pentru Boli Cardiovasculare Prof. Dr. C.C Iliescu, Soseaua Fundeni 258, Bucharest 022322, Romania; Department of Cardiology, Istanbul University-Cerrahpasa Institute of Cardiology, Haseki, Aksaray, Istanbul 34350, Turkey; Department of Cardiology, Mater Dei Hospital, Birkirkara Bypass, Triq Dun Karm, Msida 2090, Malta; Institute for Cardiovascular Diseases Dedinje, Faculty of Medicine, University of Belgrade, Heroja Milana Tepica 1, Belgrade 11040, Serbia; Department of Cardiac Surgery, Tartu University Hospital, Puusepa St. 8, Tartu 50406, Estonia; Cardiosurgery Department, Timofei Mosneaga Republican Clinical Hospital, Nicolae Testemitanu St. 29, Chisinau 2025, Moldova; Pediatric Cardiology Clinic, Paedi Center for Specialized Pediatrics, Athalassa Avenue, 178, Nicosia 2025, Cyprus; Medical Faculty Skopje, University Clinic of Cardiology, Mother Teresa 17, Skopje 1000, North Macedonia; 1st Cardiology Department, School of Medicine, Faculty of Health Sciences, Aristotle University of Thessaloniki, St. Kyriakidi 1, Thessaloniki 54124, Greece; Clinic of Cardiology, Clinical Center Banja Luka, Faculty of Medicine, University of Banja Luka, Dvanaest Beba bb St, Paprikovac, Banja Luka 78000, Bosnia and Herzegovina; Clinic of Cardiology, University Clinical Centre of Kosovo, Medical Faculty, University of Prishtina, Lagjia e spitalit, Prishtine 10000, Kosovo; Department of Congenital Heart Diseases in Children and Adults, Clinic of Cardiology, ‘St. Ekaterina’ University Hospital, Medical University Sofia, Pencho Slaveykov St. 52A, Sofia 1431, Bulgaria; Institute of Cardiology, the Adult Congenital Heart Disease Centre at the Jagiellonian University, Jagiellonian University Medical College, Anny St. 12, Kraków 31008, Poland; Ukrainian Children’s Cardiac Center, Yuriia Illienka St. 24, Kyiv 04050, Ukraine; Ukrainian Children’s Cardiac Center, Yuriia Illienka St. 24, Kyiv 04050, Ukraine; Adult Congenital Heart Centre and National Centre for Pulmonary Hypertension, Royal Brompton & Harefield Hospitals, Guys & St Thomas’s NHS Trust, Sydney Street, London SW3 6N5, UK; National Heart & Lung Institute, Imperial College, Cale Street, London SW3 6LY, UK; Adult Congenital Heart Centre and National Centre for Pulmonary Hypertension, Royal Brompton & Harefield Hospitals, Guys & St Thomas’s NHS Trust, Sydney Street, London SW3 6N5, UK; National Heart & Lung Institute, Imperial College, Cale Street, London SW3 6LY, UK; Department of Medical Rehabilitation, Medical Faculty, University of Rijeka, Ul. Braće Branchetta 20/1, Rijeka 51000, Croatia

**Keywords:** Congenital heart disease, Transition of care, Structured transition programme, Barriers to transition, Survey study

## Abstract

**Aims:**

Transition from paediatric to adult congenital heart disease (ACHD) care is a critical determinant of lifelong disease management for individuals with congenital heart disease (CHD). However, structured transition programmes remain inconsistently implemented, and data are lacking on current practices in Central and South-Eastern European (CESEE) countries. This study aimed to evaluate existing transition practices across CESEE and to identify opportunities to improve the development and implementation of structured programmes.

**Methods and results:**

An online questionnaire was distributed to centres involved in CHD care across CESEE countries. The survey assessed centre characteristics, transition services, transfer processes, and development priorities. Responses from 60 healthcare CHD professionals representing 43 centres in 21 countries were analysed. A formal ACHD service was reported in 29 (67%) of 43 centres, mostly established after the year 2000 (72%), caring for a median of 1500 (interquartile range 782–4000) patients with ACHD. Overall, only 10 (23%) centres reported having a formal transition programme. Transition counselling was typically delivered in a single outpatient visit, commonly involving paediatric and adult CHD physicians, but rarely nurses and psychosocial professionals (20% each). Structured transfer processes were inconsistently implemented, and only half of the centres monitored non-attendance at initial adult visits. In centres without formal programmes, informal counselling largely occurred at the final paediatric or first adult visit, relying on patient self-referral, and lacking follow-up.

**Conclusion:**

Formal transition programmes remain limited across CESEE ACHD centres. Standardized clear pathways, investment in dedicated transition clinics, and trained workforce are needed to improve continuity of care for adolescents with CHD.

## Graphical Abstract

**Graphical Abstract:**
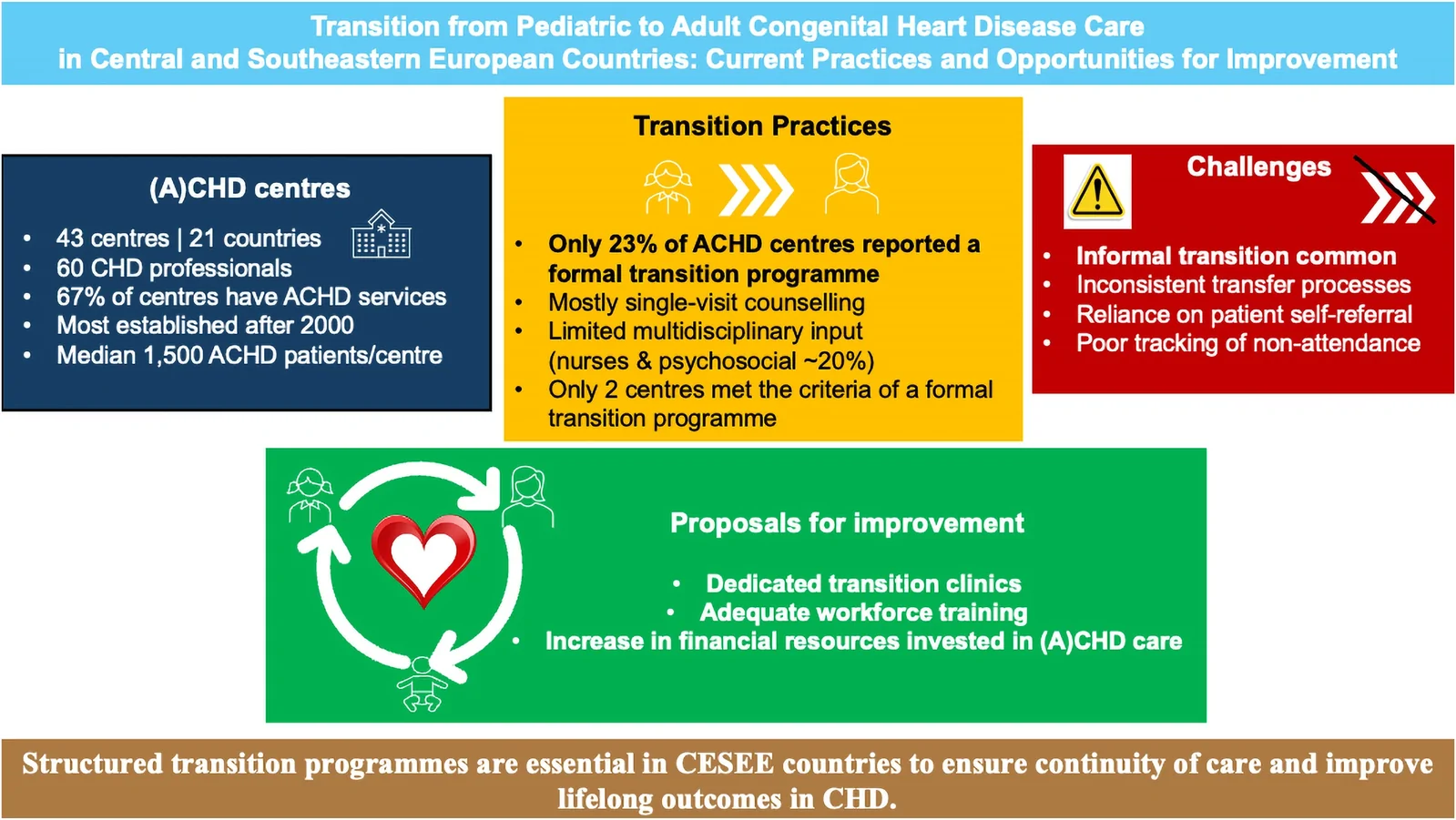

What is already known?

- People born with congenital heart disease need lifelong medical care for optimal long-term outcomes.
- Transition from paediatric to adult congenital heart disease care represents a critical period that is important for ensuring continuity, maintaining treatment adherence, and avoiding loss to follow-up. Data on current practices in Central and South-Eastern European (CESEE) countries are lacking.
- Unsuccessful transition and loss to follow-up are associated with adverse clinical outcomes.

What does this study add?

- Across 43 institutions providing congenital heart disease care in 21 CESEE countries, only 23% of centres report having a formal transition programme in place, with only two meeting the full definition criteria.
- In centres without formal programmes, the transition process consisted of informal counselling without clear planning or follow-up; patients were expected to arrange their own transfer to adult care.
- These findings document a lack of standardized, structured transition protocols across the CESEE region for patients moving from paediatric to adult congenital heart disease care.

## Introduction

Advances in the diagnosis, medical management, and surgical treatment of patients with congenital heart disease (CHD) have significantly improved survival rates, resulting in a growing population of adolescents and adults living with CHD worldwide.^1^ As a result, lifelong specialized cardiac care is essential. The transition from paediatric to adult congenital heart disease (ACHD) care represents a critical period to ensure continuity, maintain treatment adherence, and avoid loss to follow-up, which has been associated with adverse clinical outcomes.^2^

International societies, including the European Society of Cardiology and the American College of Cardiology, recommend a structured transition process to guide adolescents with CHD from paediatric to adult-oriented care.^3,4^ Effective programmes typically involve multidisciplinary teams, patient education, and coordinated transfer protocols that promote patient autonomy and sustained engagement with ACHD services. Across Western Europe, as well as in the UK and the USA, such transition programmes are increasingly established and functioning, although their coverage varies. European data, derived predominantly from Western European centres, showed that among 96 ACHD centres, 42% provided formal transition programmes and almost 90% offered structured transfer,^5^ while reports from the UK and USA similarly show that a substantial proportion of centres have implemented organized and successful transition pathways, 49% and 66%, respectively.^6,7^ Overall, despite differences in reported transition rates, these healthcare systems generally have structured transition frameworks in place.

In contrast, there is limited information regarding the availability, organization, and effectiveness of transition programmes in Central and South-Eastern European (CESEE) countries, where healthcare systems and CHD care structures may differ substantially from those in Western Europe.^8,9^ This study aimed to evaluate current transition practices from paediatric to ACHD care across CESEE, with a focus on the presence of structured programmes, organizational approaches, and perceived challenges in implementation, with the goal to identify current status, existing gaps, and opportunities for improving continuity of care for young people with CHD.

## Methods

We conducted a cross-sectional survey of healthcare professionals involved in CHD care across CESEE between September 2025 and January 2026 to assess current transition practices from paediatric to ACHD care.

A 37-item online questionnaire was developed specifically for this study by two authors (A.A. and M.B.) and was subsequently reviewed by a third author (K.P.) to ensure clarity and comprehensiveness. The survey comprised six sections: (i) characteristics of the ACHD centre; (ii) characteristics of the responding healthcare professional; (iii) general transition questions; (iv) formal transition services; (v) informal transition practices (for centres without a formal programme); (vi) transfer processes; and (vii) proposals for improving transition services. The questionnaire included yes/no, multiple-choice, numerical, single-word, and open-ended questions (see Supplementary material online, *Table S1*).

The survey was initially distributed electronically to lead physicians specializing in (A)CHD care who were affiliated with the European Society of Cardiology (ESC) CESEE Study Group (*n* = 25).^10^ To ensure broader reach, we employed a convenience and snowball sampling strategy, requesting these initial recipients to forward the survey link to relevant multidisciplinary team members within their own institutions (encouraging multiple responses per centre), as well as to lead physicians at non-affiliated CHD centres, within their respective CESEE countries. Tertiary centres across all countries within the CESEE region were invited, except for Albania, Montenegro, and Belarus, where no formal contact with a lead physician was available. Centres were eligible if they: (i) employed at least one physician with (A)CHD involvement and (ii) had at least 150 (A)CHD patients in active follow-up. Cross-validation of the participating centres was performed by the first and last authors via electronic correspondence with all respondents. Of 50 centres initially contacted or reached via forwarding, a total of 43 specialized (A)CHD centres across 21 European countries fully met the eligibility criteria and were included in the final analysis (*Figure 1*). Due to the snowball distribution method, the exact number of secondary recipients who declined or did not respond cannot be precisely quantified. Responses were anonymized at the individual level, although participants reported their professional role and institution.

**Figure 1:**
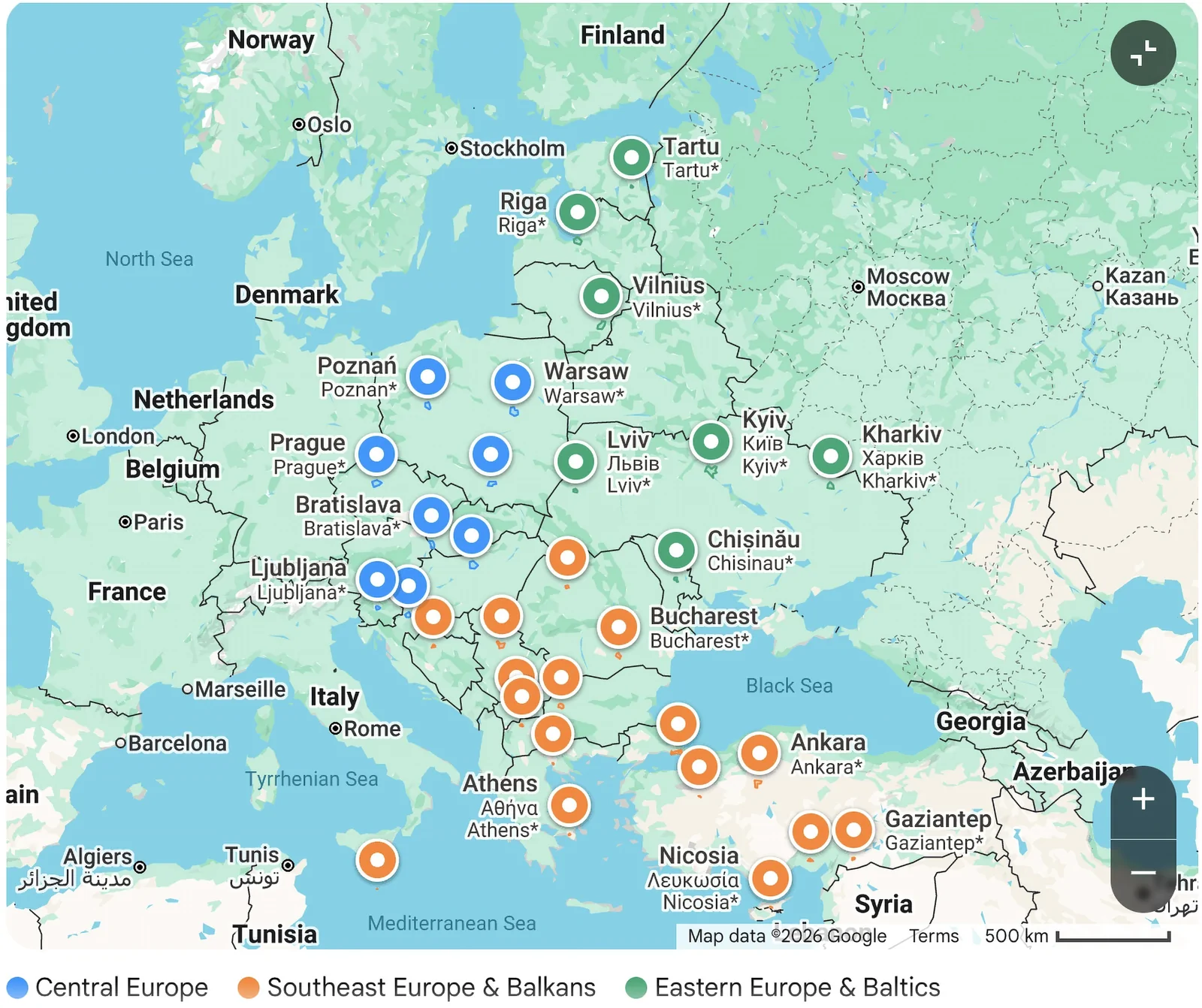
A map of Central and Southeastern European Countries and Cities with tertiary congenital heart disease centres that participated in the survey. A geographical map of Europe highlighting the 21 participating Central and Eastern European countries, with markers indicating the locations of the 43 participating congenital heart disease institutions.

A formal transition programme was defined as a structured, coordinated, and multidisciplinary preparatory process facilitating transfer from paediatric to adult care, typically including planned transition activities, patient education, and dedicated transition visits (see Supplementary material online, *Table S1*, question #17).^4,6,7^ Conversely, informal transition refers to ad-hoc, physician-led counselling delivered during routine paediatric or adult CHD visits, without a standardized protocol or tracking framework. While healthcare transition represents the longitudinal, preparatory phases to foster autonomy in CHD patients, transfer of care is defined as the discrete event marking the formal relocation of clinical responsibility from paediatric cardiology to an ACHD specialist team.^11^ Centres without a formal programme provided information on informal transition practices and transfer processes.

The study was conducted in accordance with the principles of the Declaration of Helsinki. As this study was considered a service evaluation and did not involve patient participation, formal ethical approval was not required.

### Statistical analysis

Statistical analyses were performed using IBM SPSS Statistics version 24.0 (IBM Corp., Armonk, NY, USA). Categorical variables are presented as absolute numbers (percentages), while continuous variables are reported as median ± interquartile range (IQR). In centres with more than one respondent, continuous variables were analysed at the participant level. For categorical variables with discordant responses among participants from the same centre, results were expressed as absolute numbers and percentages based on the total number of respondents. When no discrepancies were observed among respondents from the same centre, analyses were conducted at the centre level. The level of analysis (participant-level or centre-level) for each variable is outlined in Supplementary material online, *Table S1*; 12 of 37 variables were analysed at the participant level and 3 variables at both levels to demonstrate the discrepancy in perception regarding the organization of transition practices even among individuals working at the same centre. A sensitivity analysis, based on the definition of formal transition programmes employed in this survey, was also performed (see Supplementary material online, *Table S1*).

## Results

### Institutions and participants

A total of 60 healthcare professionals from 43 institutions providing CHD services across 31 cities in 21 countries completed the survey (*Table 1*). Over half of the CHD institutions (*n* = 22, 51.2%) served a population exceeding 2 million people.

**Table 1:** Representation of CESEE countries, cities, and institutions participated in the survey

Country (*n* = 21)	City (*n* = 31)	Institutions by country (*n* = 43)	Number of participants by country (*n* = 60)
**Bosnia and Herzegovina**	Banja Luka	1	1
**Bulgaria**	Sofia	2	3
**Czech Republic**	Prague	2	2
**Croatia**	Zagreb	1	3
**Cyprus**	Nicosia	1	1
**Estonia**	Tartu	1	1
**Greece**	Thessaloniki, Athens	7	10
**Hungary**	Budapest	2	2
**Kosovo**	Pristina	1	1
**Latvia**	Riga	2	2
**Lithuania**	Vilnius	2	5
**Malta**	Msida	1	1
**North Macedonia**	Skopje	1	1
**Poland**	Warsaw, Krakow, Poznan	4	5
**Republic of Moldova**	Chisinau	1	1
**Romania**	Bucharest, Cluj-Napoca	2	2
**Serbia**	Belgrade	1	1
**Slovakia**	Bratislava	1	5
**Slovenia**	Ljubljana	1	4
**Türkiye**	Ankara, Istanbul, Kütahya, Adana, Gaziantep	5	5
**Ukraine**	Kyiv, Lviv, Kharkiv	3	4

ACHD, adult congenital heart disease; CESEE, Central and Southeastern European Countries.

A formal ACHD centre was reported in 29 of 43 institutions (67%); while the remaining institutions provided mainly paediatric CHD care. Of established ACHD centres, 21 (72%) of 29 centres were established after the year 2000, with the median year of establishment being 2008 (IQR 2002–2012), based on responses from 46 participants. The median number of patients under adult care and the median number of patients with CHD under paediatric care were 1500 (IQR 782–4000) and 1993 (IQR 500–5750), respectively. The number of patients who were transferred yearly from paediatric to adult CHD care was 40 (IQR 20–130), based on responses from 55 participants.

Most participants in the survey were either cardiologists with expertise in ACHD care (20, 33%) or general adult cardiologists (17, 28%), and worked in an ACHD service (40, 67%) (*Table 2*). One-quarter of participants (15, 25%) were from a strictly paediatric background, including paediatric CHD specialists (13, 21.6%) and paediatric CHD fellows (2, 3.2%). About one-fifth of participants (11, 18.3%) cared for both paediatric and adult patients with CHD.

**Table 2:** Participants’ profession and sector of employment

Number of participants	*Ν* = 60
**Participants’ profession**	
** Cardiologist with expertise in ACHD**	20 (33.3%)
** General** c**ardiologist**	17 (28.3%)
** Paediatric CHD** s**pecialist**	13 (21.6%)
** ACHD** f**ellow**	3 (5%)
** Nurse with experience in (A)CHD**	3 (5%)
** Paediatric CHD** f**ellow**	2 (3.2%)
** Cardiology** t**rainee**	1 (1.6%)
** Social** w**orker**	1 (1.6%)
** Clinical** p**sychologist**	—
** Administrative staff**	—
**Sector of employment**	
** ACHD centre**	40 (66.6%)
** Paediatric CHD centre**	9 (15%)
** Both**	11 (18.3%)

ACHD, adult congenital heart disease; CHD, congenital heart disease.

### Formal transition programme

Overall, of the 43 participating centres caring for CHD patients, 10 (23%) reported having a formal transition programme. Among these, transition counselling mostly consisted of a single, on-site individual visit (6/10, 60% of formal transition programmes). One-third of programmes provided counselling through a series of up to three on-site outpatient visits, while one centre offered counselling in one or more group sessions.

In the majority of programmes (6/10, 60%), counselling was delivered through a joint service involving both paediatric and adult CHD providers; a paediatric CHD specialist was involved in 90% of programmes, and a cardiologist with expertise in ACHD care in 80%. A nurse with CHD experience participated in 40% of programmes, while access to a psychologist or social worker was limited to 20%. The remaining one-third of programmes were conducted within paediatric outpatient services, and one programme was conducted in an ACHD outpatient service. Among participants working in institutions with a formal transition process, 60% (9/15) reported counselling visits lasting more than 30 min, while 20% (3/15) reported durations of 15–30 min and 20% (3/15) reported durations of less than 15 min. Based on a sensitivity analysis using the definition for formal transition programmes as stated in this survey (see Supplementary material online, *Table S1*), only two centres reported transition managed jointly by paediatric and ACHD teams during serial visits lasting over 15 min. Additionally, only one centre utilized a fully staffed transition team—including a nurse with experience in CHD, a psychologist, and a social worker alongside CHD physicians. At this centre, on-site counselling was provided once, and visits lasted more than 30 min.

In 80% (8/10) of centres with formal transition programmes, transition to adult care occurred between the ages of 18 and 20 years. Furthermore, most centres with a formal programme (80%) discussed high-risk adolescents transitioning to adult care in multidisciplinary team meetings. All educational activities that were reported to be included in formal transition counselling programmes are presented in *Table 3*. Only two centres used a patient questionnaire, administered after the transition visit, to assess patients’ understanding of their condition and their knowledge regarding preventive measures.

**Table 3:** Educational activities provided in 10 formal transition programmes as reported by 15 participants

**Patients’ understanding about their primary CHD diagnosis**	**13 (86.6%)**
**Patients’ understanding regarding previous surgical/interventional procedures**	13 (86.6%)
**Patients’ understanding regarding potential long-term complications associated with their disease**	13 (86.6%)
**Patients’ understanding about their estimated prognosis**	12 (80%)
**Patients’ education about infective endocarditis (signs, symptoms, preventive measures)**	13 (86.6%)
**Patients’ counselling about reproduction (contraceptive measures, pregnancy, surrogacy**, **etc)**	12 (80%)
**Patients’ education about** a **healthy diet and exercise (leisure and/or competitive sports)**	13 (86.6%)
**Patients’ counselling about career options in relation to their level of disability**	10 (66.6%)
**Other**	1 (10%)
**Missing response**	1 (10%)

CHD, congenital heart disease.

Regarding the process of scheduling the patient’s first appointment with the ACHD team, in half of the centres with a formal transition programme, paediatric CHD team refers patients to the ACHD service, after which the ACHD team schedules the appointment and informs patients. In 40% of centres, appointments were coordinated through a joint paediatric CHD and ACHD service, which is responsible for both scheduling and patient communication. A formal process to verify successful transfer to the ACHD service, including confirmation that the patient was scheduled for an initial ACHD clinic visit, was in place in 70% of these centres (conducted either by a physician, or a nurse or administrative staff), while half of the centres with a formal transition programme tracked patients who did not attend their first ACHD outpatient appointment, to contact them and reschedule.

### Informal transition process

Among institutions without a formal transition programme (33/43, 77%), informal counselling usually took place either at the paediatric CHD outpatient clinic during the last visit (23/33, 70%) or/and at the ACHD outpatient clinic during the first visit (27/33, 82%). Among centres without a formal transition programme, 48% of participants (22/45) reported that informal counselling lasted more than 30 min, 22.2% (10/45) reported durations of 15–30 min, and fewer than 9% reported durations of less than 15 min. Educational activities provided during this informal counselling are listed in *Table 4*.

**Table 4:** Educational activities provided during informal counselling as reported by 45 participants from 33 institutions without a formal transition programme

**Patients’ understanding about their primary CHD diagnosis**	**36 (80%)**
**Patients’ understanding regarding previous surgical/interventional procedures**	33 (73.3%)
**Patients’ understanding regarding potential long-term complications associated with their disease**	39 (86.6%)
**Patients’ understanding about their estimated prognosis**	25 (55.5%)
**Patients’ education about infective endocarditis (signs, symptoms, preventive measures)**	39 (86.6%)
**Patients’ counselling about reproduction (contraceptive measures, pregnancy, surrogacy**, **etc)**	36 (80%)
**Patients’ education about** a **healthy diet and exercise (leisure and/or competitive sports)**	33 (73.3%)
**Patients’ counselling about career options in relation** to **their level of disability**	19 (42.2%)
**Other**	5 (11.1%)
**Missing response**	4 (8.9%)

CHD, congenital heart disease.

In centres without an established transition programme, the majority of patients were discharged from paediatric service between 18 and 20 years of age (20/33, 61%). More than one-third of participants (16/45, 36%) stated that patients need to self-transfer and self-refer to the ACHD service. One third of participants (15/45, 33%) reported that the paediatric CHD team refers the patient to the ACHD service and the ACHD team books the appointment and informs the patient, while only a minority (5/45, 9%) indicated a joint paediatric and ACHD service that books the appointments and informs the patients. A process to verify successful transfer to the ACHD service, including confirmation that the patient was scheduled for an initial ACHD clinic visit, was reported by only 24.4% of participants (11/45), led mainly by physicians (7/11). Moreover, fewer than half of centres (15/33, 45.5%) without a formal transition programme reported tracking patients who miss their first ACHD outpatient appointment and contacting them to arrange a new appointment.

### Priorities in establishing a successful transition programme

All participants were asked to identify three main factors they considered most important for establishing a successful structured transition CHD programme. The most frequently reported priorities were adequate staffing and appropriate staff training (92%), followed by improved financial resources (50%) and a dedicated weekly transition clinic (42%) (*Table 5*).

**Table 5:** Factors that promote the organization of a successful transition programme as reported among 60 participants from 43 (A)CHD centres

Adequate number of staff and adequate staff education	**55 (91.6%)**
Financial resources	30 (50%)
A weekly dedicated transition clinic	25 (41.6%)
Technological support	22 (36.6%)
Changes in government’s policies	21 (35%)
Optimally, three transition visits per patient with the last including a survey of patient’s understanding of the educational activities provided	19 (31.6%)

CHD, congenital heart disease.

## Discussion

In this multinational, multicentre survey, we investigated transitional practices between paediatric and adult CHD services in CESEE countries. The main findings demonstrated that fewer than a quarter of centres caring for patients with CHD had a self-reported formally established transition programme, with only two centres meeting the strict definition criteria for a formal transition programme. Most self-reported formal programmes consisted of a single transition visit lasting more than 30 min and involving both paediatric and adult consultants. Formal programmes demonstrated relatively high levels of transfer verification and patient tracking (70%). In contrast, the majority of CESEE centres (77%) lacked a structured transition process, requiring patients to self-transfer and independently engage with ACHD services. This is frequently accompanied by the absence of transfer verification and limited or no tracking of patients who fail to attend their first adult service appointment.

The proportion of centres with formal transition programmes is significantly lower than the European average,^5^ despite the increasing recognition of transition as a crucial component of lifelong care for patients with CHD.^1,12^ Most ACHD centres in CESEE countries were established after the year 2000, potentially reflecting the relatively recent and ongoing development of specialized services in these countries compared with the more established ACHD services in Western European countries.^3^ This gap may also reflect competing resource demands, limited workforce capacity, and variable institutional prioritization.^10^

Where formal programmes were available, most were based on a single session. However, transition should ideally be a multiyear process of education and preparation for adult life with CHD rather than a one-time clinic transfer.^6^ Education on individual CHD trajectories, potential complications, and the importance of early lifestyle choices should ideally begin at diagnosis and involve parents or caregivers, with the patient progressively engaged from childhood through adolescence into young adulthood.^11,13^ Digital tools and open access to electronically published data can facilitate this process and should be leveraged more widely.^14^

Encouragingly, the single transitional visit involved both paediatric cardiologists and cardiologists with expertise in ACHD care. However, there was limited participation of allied health professionals, including nurses, psychologists, and social workers, suggesting that the recommended multidisciplinary approach remains restricted.^11,15^ This is relevant, as transition extends beyond medical management to encompass psychosocial support, education, and the promotion of self-management skills, areas where multidisciplinary input is essential.^15^

In most centres, transition started between 18 and 20 years. Consensus documents and guidelines propose that transitional care should begin earlier in adolescence, at 14–15 years of age, aiming to transition into ACHD care in young adulthood, and continue education beyond that transition into emerging adulthood.^4,11^ Therefore, centres across CESEE countries should aim to initiate the transition process earlier to improve long-term effectiveness and reduce the loss of continuity of care into adulthood.^16^ The timing of transition should be guided by emotional maturity and the developmental level for transition planning and should be tailored to each patient’s needs.

Most transition visits lasted at least 30 min; however, very few centres reported using structured assessment tools, such as patient questionnaires, to evaluate understanding and readiness. Hence, the content and delivery of transition counselling cannot be adequately measured. Moreover, this lack of standardized evaluation may hinder the ability to tailor interventions to individual patient needs and to measure programme effectiveness.^7^ Furthermore, while multidisciplinary discussions for high-risk patients were common in centres with formal programmes, systematic approaches to monitoring transition success, such as attendance at the first ACHD visit, were less consistently implemented.

Importantly, our study showed that most CESEE countries and specialized centres lacked formal transition programmes, relying heavily on informal practices, which typically occured at the final paediatric visit or the first adult clinic encounter. Although the duration of these interactions was often similar to that of formal sessions, the absence of structured processes was evident in key areas, notably in need for young patients to self-refer and self-transfer to ACHD care, and in lack of established mechanisms to verify successful transfer or track non-attendance. These findings are concerning, as loss to medical follow-up during the transition period is a well-documented risk associated with adverse clinical outcomes in CHD population.^16–18^

These findings do not indicate deficiencies in individual clinical practice but rather highlight structural limitations across the CESEE region in the organization of transition services. Much of the work undertaken by healthcare personnel remains informal and reactive, often occurring in response to patient-initiated contact. In the absence of formal transition programmes with clearly defined structures, service demand is likely underestimated, making it challenging to plan and implement effective models for improving CHD care. Addressing this gap is therefore essential for the future development of CHD services. This study should serve as a starting point for stakeholders to advocate for appropriate resources in the region, including adequate staffing, targeted training, and the establishment of dedicated transition clinics supported by sustainable funding frameworks, ultimately aiming to improve long-term CHD outcomes and reduce loss to follow-up.^19–21^ The establishment of dedicated transition clinics should be a key priority in CESEE countries. This highlights the importance of allocating protected time and creating structured environments to ensure transition activities are delivered consistently and effectively.

Furthermore, an important issue in ACHD care is the lack of a uniform, formal subspecialty certification for ACHD across many European nations, including the participating CESEE countries. In this study, ‘cardiologists with expertise in ACHD care’ were identified based on self-reported primary clinical dedication or training in ACHD care rather than standardized national credentials. This heterogeneity highlights a broader systemic challenge: the critical need for qualified ACHD cardiologists and specialized (A)CHD nurses, supported by structured, standardized regional or European education and certification pathways.^22^ Addressing this educational gap is fundamental to achieving consistent, high-quality care throughout the patient transition process.

### Study limitations

The findings of this study must be interpreted within the context of several limitations. First, the modest sample size, although geographically diverse, may impact the generalizability of our results. Second, voluntary participation and the exclusion of ‘low-volume’ centres—particularly in rural areas, may introduce a potential selection bias. Third, the underrepresentation of allied health professionals may be attributed to our survey distribution method, as well as the limited involvement of these professionals in the transition of care, overall. Fourth, the self-reported nature of the data may subject them to recall or reporting bias. Additionally, responses from multiple participants within the same centre were not always concordant, reflecting either differences in individual perceptions or a lack of formal documentation of patient services provided. To account for these differences, a participant-level analysis was conducted, introducing a potential risk of overrepresentation bias from centres with multiple participants; however, such centres constituted a minority. Finally, this study is descriptive and utilizes a cross-sectional design, which precludes the assessment of causal relationships or longitudinal outcomes. Therefore, an evaluation of the long-term clinical efficacy of organized transition programmes remains outside the scope of the current work and represents a critical area for future research, as the external validity of existing literature on transition programmes may be constrained when applied to the unique sociopolitical contexts examined here, highlighting the need for context-specific future studies.

### Conclusion

In conclusion, this multinational study highlights substantial gaps in the organization of transition services for patients with CHD among CESEE countries, primarily driven by structural rather than individual practice limitations. While a small proportion of centres have established formal transition programmes, often providing appropriately structured and multidisciplinary care, most centres continue to rely on informal, unstructured processes that place the burden of transition on patients and risk loss to follow-up. Future research should focus on recording loss-to-follow-up rates, first adult visit attendance, and transition-related adverse events among patients with CHD in these countries, to better evaluate the impact of healthcare system deficiencies on clinical outcomes. Additionally, the current survey underscores the urgent need to develop formalized transition pathways, including dedicated clinics, protected clinical time, and systematic patient tracking. Addressing these priorities through targeted investment in staffing, training, and sustainable service models will be essential to improve continuity of care and long-term outcomes for patients with CHD across the CESEE region.

## Supplementary Material

oeag144_Supplementary_Data

## Data Availability

The data underlying this article will be shared on reasonable request to the corresponding author.
