# Supplementary material for: Transition from paediatric to adult congenital heart disease care in Central and South-Eastern Europe: current practices and opportunities for improvement
Source: Eur Heart J Open. 2026 Sep 26;6(5):oeag144. doi: 10.1093/ehjopen/oeag144 (PMC13663801; doi:10.1093/ehjopen/oeag144)
Supplement: Supplementary file 1 [file oeag144_supplementary_data.docx]

**Supplementary Table 1.** Overview of the 37-item survey filled in by congenital heart disease healthcare providers across 21 Central and Southeastern European Countries.

| **Survey items** | **Type of answers** | **Level of statistical analysis** |
| --- | --- | --- |
| A. **Information about the institution and CHD center** | |  |
| 1. In which country do you work? | Single-word answer | Centre Level |
| 2. In which city do you work? | Single-word answer | Centre Level |
| 3. What is the name of your institution? | Short answer | Centre Level |
| 4. Do you have an established adult congenital heart disease (ACHD) centre in your institution? | Yes/No answer | Centre Level |
| 5. If the answer to question #4 is yes, approximately what year was the ACHD centre established? | Number | Participant Level |
| 6. If the answer to question #4 is yes, who leads the ACHD program? (Please provide the name of the physician) | Short answer | Centre Level |
| 7. If the answer to question #4 is yes, what is the average number of ACHD patients treated at your center? | Number | Participant Level |
| 8. Do you have a pediatric CHD center in your institution? | Yes/No answer | Centre Level |
| 9. If the answer to question #8 is yes, who leads the pediatric CHD program? (Please provide the name of the physician) | Short answer | Centre Level |
| 10. If the answer to question #8 is yes, what is the average number of pediatric CHD patients treated at your center? | Number | Participant Level |
| 11. Is there both an ACHD center and a pediatric CHD center located within the same hospital? | Yes/No answer | Centre Level |
| 12. What is the estimated size of the population served by your CHD center. A population of: | Multiple choice answer   - < 1 million - 1-2 million - 2-3 million - >3 million | Centre Level |
| **B. Details about the healthcare provider completing the survey** | |  |
| 13. What is your profession? | Multiple choice answer   - Cardiologist with expertise in ACHD care - Pediatric CHD Specialist - ACHD Fellow - Pediatric CHD Fellow - General Cardiologist - Cardiology Trainee - Nurse with experience in (A)CHD care - Clinical Psychologist - Administrative Staff - Social Worker - Other | Participant Level |
| 14. In which sector do you work? | Multiple choice answer   - ACHD centre - Paediatric CHD centre - Both ACHD and Paediatric CHD centre | Participant Level |
| **C. General transition questions** | |  |
| 15. At what age does the transition usually occur at your institution? | Multiple choice answer   - < 18 years old - 18-20 years old - >20 years old | Centre Level |
| 16. Do you hold a multidisciplinary meeting to discuss high-risk young patients transitioning from pediatric to adult care? | Yes/No answer | Centre Level |
| **D. Formal transition facilities** | |  |
| 17.  Do you have a formally established program for transitioning patients with CHD from pediatric to adult care? (A formal transition CHD program is a structured, organized process designed to facilitate the seamless transfer of patients with congenital heart conditions from pediatric to adult healthcare services. This program typically includes coordinated planning, education, and support to ensure continuity of care, promote patient independence, and address the medical, psychological, and social needs of young adults with congenital heart disease most commonly via 2-3 transition clinical appointments led by doctors or/and nurses. It aims to optimize health outcomes by preparing patients for adult healthcare management and fostering effective communication between pediatric and adult care teams.) | Yes/No answer | Centre Level |
| 18. If the answer to question #17 is yes, who is involved in the transition program? (Please select all options that apply). | Multiple choice answer   - Nurse with experience in (A)CHD care - Cardiologist with expertise in ACHD care - Paediatric CHD Specialist - Trainee doctor - Psychologist - Social Worker - Other | Centre Level |
| 19. If the answer to question #17 is yes, where does the transition clinic take place? | Multiple choice answer   - In the paediatric CHD outpatient service - In the ACHD outpatient service - There is a joint service for paediatric and adult CHD | Centre Level |
| 20. If the answer to question #17 is yes, how is the transition counselling provided? | Multiple choice answer   - Transition counselling takes place once on-site on an individual basis - Serial transition counselling visits take place (1-3 on-site outpatient visits) on an individual basis - Transition counselling is held in group sessions (1 or more sessions) - Transition counselling is provided once remotely (e.g. via teams, zoom or telephone) - Transition counselling is provided as a hybrid approach (both on-site and remote counselling) | Centre Level |
| 21. If the answer to question #17 is yes, by whom is it led? | Multiple choice answer   - ACHD team - Paediatric CHD team - Nurse led - Both ACHD and paediatric CHD team | Centre Level |
| 22. If the answer to question #17 is yes, which educational activities are included? (Please select all that apply). | Multiple choice answer   - Patients’ understanding about their primary CHD diagnosis - Patients’ understanding regarding previous surgical/interventional procedures - Patients’ understanding regarding potential long-term complications associated with their disease - Patients’ understanding about their estimated prognosis - Patients’ education about infective endocarditis (signs, symptoms, preventive measures) - Patients’ counselling about reproduction (contraceptive measures, pregnancy, surrogacy etc) - Patients’ education about healthy diet and exercise (leisure and/or competitive sports) - Patients’ counselling about career options in relation with their level of disability - Other | Participant Level |
| 23.  If the answer to question #17 is yes, how much time does a transition counselling last? | Multiple choice answer   - <15 minutes - 15-30 minutes - > 30 minutes | Participant Level |
| 24. If the answer to question #17 is yes, do you provide any patient questionnaire after each transition counselling visit to understand the level of patient understanding? | Yes/No answer | Centre Level |
| 25. If the answer to question #24 is no, please describe the type of metrics you use. | Free-text answer |  |
| E. Informal transition process |  |  |
| 26. If the answer to question #17 is no (that there is no formal transition programme in your centre), please explain briefly how patients are transferred from the paediatric to the adult CHD care. | Short answer | Centre Level |
| 27. If the answer to question #17 is no, does any informal counselling take place at the paediatric CHD outpatient clinic during the last visit before transfer to the ACHD team? | Yes/No answer | Centre Level |
| 28. If the answer to question #17 is no, does any informal counselling take place at the ACHD outpatient clinic during the first visit? | Yes/No answer | Centre Level |
| 29. If the answer to question #28 is yes, please answer how much time you spent with the patient during the first visit in the adult service. | Multiple choice answer   - <15 minutes - 15-30 minutes - > 30 minutes | Participant Level |
| 30.  In case an informal counselling is offered, which educational activities does it include? | Multiple choice answer   - Patients’ understanding about their primary CHD diagnosis - Patients’ understanding regarding previous surgical/interventional procedures - Patients’ understanding regarding potential long-term complications associated with their disease - Patients’ understanding about their estimated prognosis - Patients’ education about infective endocarditis (signs, symptoms, preventive measures) - Patients’ counselling about reproduction (contraceptive measures, pregnancy, surrogacy etc) - Patients’ education about healthy diet and exercise (leisure and/or competitive sports) - Patients’ counselling about career options in relation with their level of disability - Other | Participant Level |
| **F. Transfer facilities** | |  |
| 31.  Who is in charge of booking patient's first appointment with the ACHD team? | Multiple choice answer   - GP refers the patient to the ACHD service and ACHD team books the appointment and informs the patient - Paediatric CHD team refers the patient to the ACHD service and ACHD team books the appointment and informs the patient - There is a joint paediatric CHD and ACHD service that book the appointments and inform the patient - Patients are self-transferred to the ACHD service (reach out the ACHD team themselves to book their first appointment) - Other | For formal programmes: centre level analysis  For informal programmes: participant level analysis |
| 32. Please estimate the average number of patients that are transferred from paediatric CHD to the ACHD service per year (Please type a number). | Number | Participant Level |
| 33.  Do you have any process to check that patient transfer to ACHD service was successful and that patient has been scheduled in the first ACHD clinic? | Yes/No answer | For formal programmes: centre level analysis  For informal programmes: participant level analysis |
| 34. If the answer to question #33 is yes, please explain the procedure. | Multiple choice answer   - Nurse check - Physician check - Check by administrative staff - All of the above | For formal programmes: centre level analysis  For informal programmes: participant level analysis |
| 35.  Does your ACHD centre track ‘no show’ patients during the first ACHD outpatient visit to call them and reschedule the appointment? | Yes/No answer | Centre Level |
| **G. Proposal for improving transition services** | |  |
| 36.  What is in your opinion the 3 fundamentals in order to organize a successful transition program? (multiple answers) | Multiple choice answer   - Changes in government’s policies - Financial resources - Technological support - Adequate number of staff and adequate staff education - A weekly dedicated transition clinic - Optimally, 3 transition visits per patient with the last including a survey of patient’s understanding of the educational activities provided | Participant Level |
| 37.  Please write in your own words what are the most important future measures in order to establish and run a successful transition program. | Free-text answer | Participant Level |
